# Linking Increasing Drought Stress to Scots Pine Mortality and Bark Beetle Infestations

**DOI:** 10.1100/tsw.2007.58

**Published:** 2007-03-21

**Authors:** Matthias Dobbertin, Beat Wermelinger, Christof Bigler, Matthias Bürgi, Mathias Carron, Beat Forster, Urs Gimmi, Andreas Rigling

**Affiliations:** ^1^ Swiss Federal Research Institute for Forest, Snow and Landscape Research WSL, Zürcherstrasse 111, CH-8903 Birmensdorf, Switzerland; ^2^ Forest Ecology, Institute of Terrestrial Ecosystems, Department of Environmental Sciences, ETH Zurich, CH-8092 Zürich, Switzerland; ^3^ Euloz, CH-1926 Fully, Switzerland

**Keywords:** global warming, drought, bark beetle, tree mortality

## Abstract

In the dry Swiss Rhone Valley, Scots pine forests have experienced increased mortality in recent years. It has commonly been assumed that drought events and bark beetles fostered the decline, however, whether bark beetle outbreaks increased in recent years and whether they can be linked to drought stress or increasing temperature has never been studied.

In our study, we correlated time series of drought indices from long-term climate stations, 11-year mortality trends from a long-term research plot, and mortality probabilities modeled from tree rings (as an indicator of tree vitality) with documented occurrences of various bark beetle species and a buprestid beetle, using regional Forest Service reports from 1902 to 2003 and advisory cases of the Swiss Forest Protection Service (SFPS) from 1984 to 2005. We compared the historical findings with measured beetle emergence from a 4-year tree felling and breeding chamber experiment.

The documented beetle-related pine mortality cases increased dramatically in the 1990s, both in the forest reports and the advisory cases. The incidents of beetle-related pine mortality correlated positively with spring and summer temperature, and with the tree-ring-based mortality index, but not with the drought index. The number of advisory cases, on the other hand, correlated slightly with summer drought index and temperature, but very highly with tree–ring—based mortality index. The tree-ring-based mortality index and observed tree mortality increased in years following drought. This was confirmed by the beetle emergences from felled trees. Following dry summers, more than twice as many trees were colonized by beetles than following wet summers.

We conclude that increased temperatures in the Swiss Rhone Valley have likely weakened Scots pines and favored phloeophagous beetle population growth. Beetles contributed to the increased pine mortality following summer drought. Among the factors not addressed in this study, changed forest use may have also contributed to increased beetle populations and Scots pine mortality, whereas air pollution seems to be of lesser importance.

## INTRODUCTION

Increasing temperatures during spring and summer as a result of global warming is believed to increase the likelihood of insect outbreaks in semi-arid and temperate regions. On the one hand, increasing temperature under constant precipitation should, theoretically, increase drought stress to trees[1]. On the other hand, warmer spring and summers should favor population growth of bark beetles[2]. It is less known whether increased population growth of antagonists or insect pathogens may counteract this trend or if trees are able to adapt.

Recently, large-scale outbreaks of bark beetles have been reported from Alaska[3], British Columbia[4], and large areas of the southwestern U.S.[5]. In France, Switzerland, and southern Germany, increased tree mortality and bark beetle outbreaks have also been observed in recent years[6]. In the North American cases, mortality and bark beetle outbreaks followed extended or severe drought periods and in the European cases, bark beetle outbreaks followed large-scale storm damages in 1999 and the severe drought in 2003. The question remains if these outbreaks have increased in magnitude due to global warming.

In this study, we investigate relationships between climate variability, tree mortality, and bark beetle infestations in the Swiss Rhone Valley. In this dry valley, Scots pine forests have experienced increasing mortality in recent years[7,8,9]. While precipitation in the research area has changed little, increasing temperature has led to a recent increase in summer drought stress, in particular in the last 25 years. Although various bark beetles and other phloeophagous beetles have usually been observed as secondary agents in single dying trees, no typical bark beetle mass infestation pattern has been observed. In this study, we will analyze whether (a) bark beetle–related tree mortality has increased, (b) beetle infestations can be linked to temperature, or (c) beetle occurrence correlates either with drought or with tree vitality.

## METHODS AND MATERIALS

We used various datasets in our studies. For beetle occurrence or outbreaks, we used historical records on Scots pine decline from forest reports from the Canton Valais, recent advisory cases related to Scots pine mortality from the SFPS of the Swiss Federal Research Institute WSL, and data from a 4-year field trial between May 2001 and February 2005. Regional and local forest management reports from 1902 to 2003 were obtained, and species-specific mortality and its causes were extracted. For this study, we used only the number of reported incidents per year that mentioned Scots pine mortality and reported beetle occurrence. The SFPS is usually called in by the local Forest Service to determine the causes of tree diseases or decline, and to discuss management practices, and results are less biased by forest policy decisions than forest reports. For this study, we used the number of advisory cases per year for all cases of pine mortality related to beetles between 1984 and 2005. During the field trial, around 450 pines from two study sites were numbered and tree crowns were visually assessed using binoculars in early May and early February. Crown transparency, assessed in 5% steps using standard photographs as a comparison, was used as tree vitality indicator (0% means a fully foliated tree, while 100% is a dead tree). Following each assessment, trees were stratified into four classes: healthy (0–20% crown transparency), slightly weakened (25–40%), severely weakened (45–80%), and moribund (85–100%). Within the three later classes, trees were randomly selected for felling. A total of 209 trees were felled. Stem sections from various tree heights were taken from each tree and placed in breeding chambers[10].

Annual tree mortality, assessed each summer in July, was available from the long-term forest ecosystem research (LWF) site Visp since 1996[1,11]. To test whether observed bark beetle occurrence was related to susceptibility of trees, we used tree rings as tree vitality indicators. Bigler and Bugmann[12] used tree rings from dead and live Norway spruce trees and fit a logistic regression model using the current relative basal area growth and the regression slope over growth of the past 5 years as input variables. A tree-ring series taken in 2001 had previously been used to calibrate the model for the Valais and the index calculated for other series collected between 1977 and 2001 in various parts of the main Rhone valley[9]. For this study, we used the mean of the mortality indices calculated for all tree-ring series.

Two official long-term climate stations of MeteoSwiss are located within our study area. The station Sion is one of 12 Swiss sites with homogenized monthly data for the time period 1864 to 2000[13]. The station Visp has precipitation records since 1901, but temperature measurements only since 1980. We homogenized precipitation data for Visp and used partially adjusted temperature data from Sion to compute the drought index for Visp[9]. In this study, we used a mean drought index of both climate stations. The monthly drought index by Thornthwaite was calculated as the difference between precipitation and potential evapotranspiration[14]. Data were either visually compared or analyzed using pair-wise Spearman’s rank correlation coefficients, which is robust to non-normality in the data.

## RESULTS

Annual mortality rates of Scots pines between 1996 and 2006 on the 2-ha LWF site Visp, that totaled more than 60% of the standing live trees, correlated highly with calculated drought stress of the previous summer (Fig. 1). Highest rates were observed following the drought summers in 1998 and 2003. Incidents of beetle-related mortality in forest reports showed two distinct peaks in the late 1940s to early 1950s and from 1990 onward (Fig. 2). Including all mortality incidents changed the trend only slightly. Advisory cases related to the five most frequent beetles *Ips sexdentatus*, *I. acuminatus*, *Tomicus minor* or *T. pinipera*, and *Phaenops cyanea* in Scots pines also increased in the late 1990s, but showed distinct peaks in 1991–92, 1998–99, and 2004–05 (Fig. 3). Correlation between advisory cases and beetle-related mortality incidents in reports between 1984 and 2003 was low. In particular, the high number of advisory cases in 1991 and 1992 was not matched by the reports. The tree-ring–based mortality index showed distinct peaks in 1921, the mid and late 1940s, the 1970s, and around 1990 and 1998 (Fig. 4). The correlations between all incidents of mortality or beetle-related mortality incidents only were highest with spring or summer temperature, lower with fall and winter temperatures (Table 1). The incidents were also highly correlated with the tree-ring–based mortality index, but not clearly with previous years or summer drought (Table 1). The high correspondence of above-average temperatures and beetle-related mortality cases is striking (Fig. 5) when comparing the time trend of beetle-related incidents with the 5-year running mean for the deviation of summer and spring temperature from the overall mean. While warmer periods also coincide with drought stress (late 1940s and the 1990s), other cool periods with drought stress, such as the mid 1960s or mid 1970s, had only few or no beetle incidents. Beetle-related advisory cases since 1984, on the other hand, correlated only slightly with temperature and previous summer drought index, but very highly with the tree-ring based mortality index (Table 1, Fig. 3). Not surprisingly, the tree-ring–based mortality index was correlated with the annual drought stress index (Spearman rank correlation coefficient of –0.36, Fig. 4). In particular during and following drought years or drought periods the tree-ring based mortality index increases drastically (Fig. 4).

Emerging beetles in the breeding chambers between 2001 and 2005 included among others *I. acuminatus*, *Tomicus* spp., and *P. cyanea* as the most frequent of the potentially noxious beetles, but almost no *I. sexdentatus*. Frequency of trees infested by these species were more than twice as high following the dry and warm summers 2000, 2003, and 2004 than following the wet summers 2001 and 2002 (significant for all species and *P. cyanea*, Table 2), while the proportion of trees with low vitality was not different between the samples. Years with high beetle occurrence coincided with high mortality rates observed at the long-term monitoring sites Visp (mean mortality following the dry summers: 17.7%, following the wet summers: 1.3%) and with the number of advisory cases (mean number of cases in years following dry summers: 12.3, years following wet summers: 1.5).

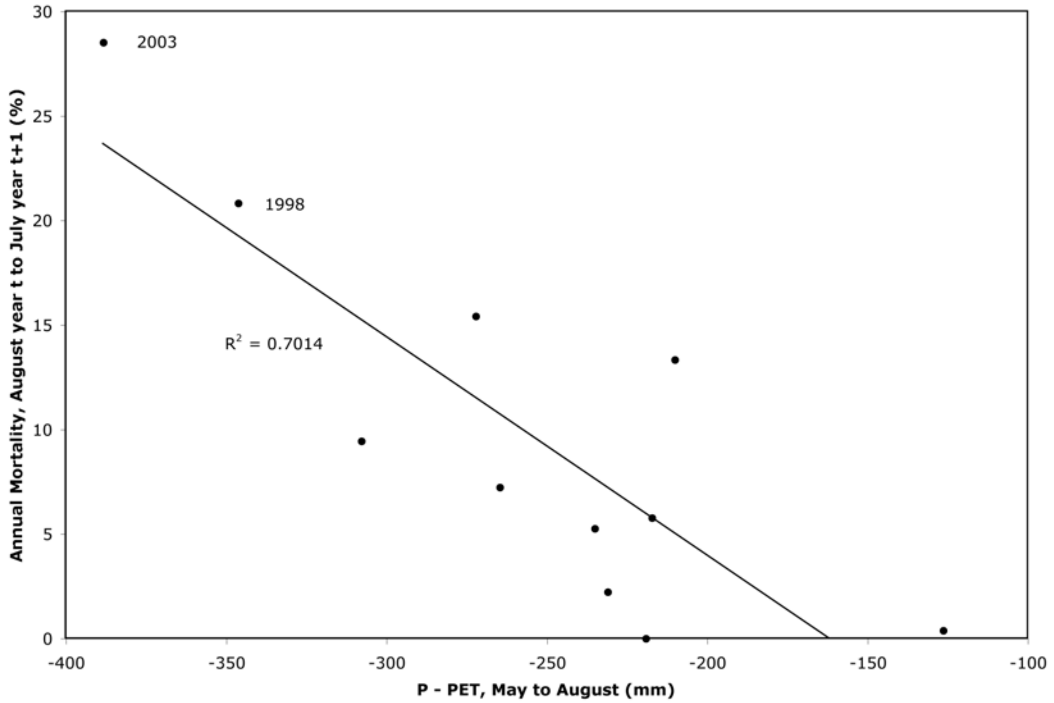

**FIGURE 1.** Annual pine mortality (assessed in late July) at the research site Visp from 1996 to 2006 against difference between mean precipitation (P) and calculated potential evapotranspiration (PET, method by Thornthwaite) during the previous May to August.

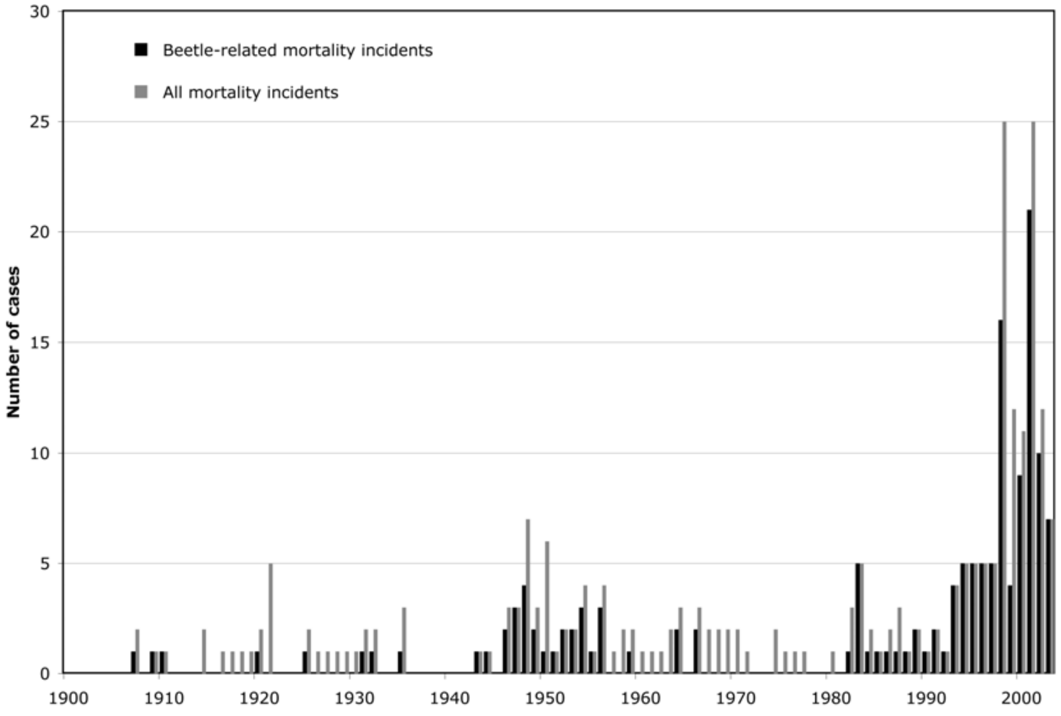

**FIGURE 2.** Incidents of Scots pine mortality in general (light grey) and mortality related to beetles only (dark grey, 1902–2003).

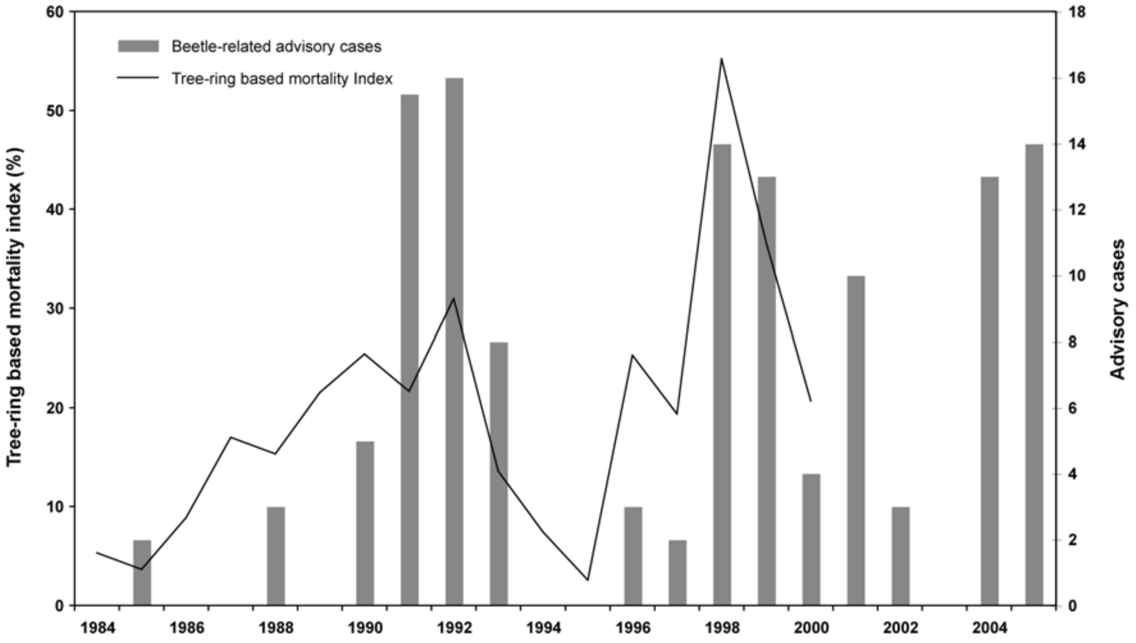

**FIGURE 3**. Number of beetle-related advisory cases and tree-ring–based mortality index (only until 2000).

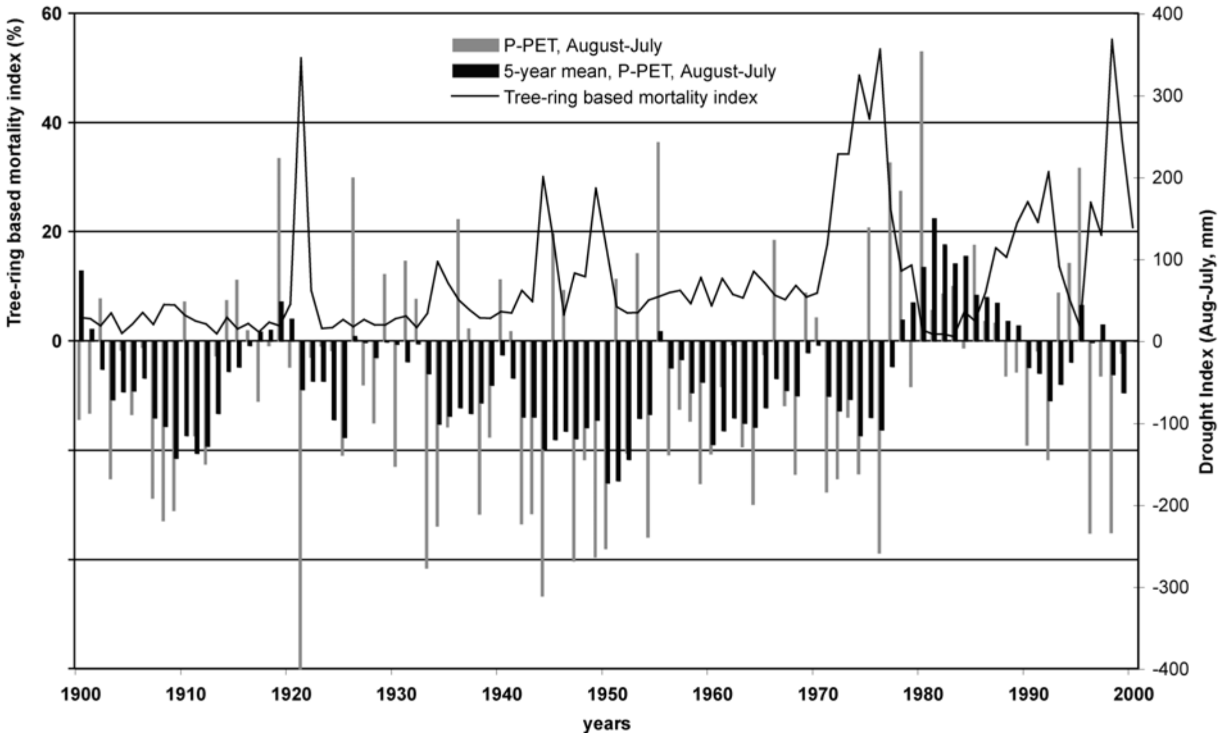

**FIGURE 4.** Tree-ring–based mortality index (see text), annual drought index (P-PET, August previous year to July current year), and 5-year running mean drought index.

**TABLE 1**
**Spearman Rank Correlations for the Time Series of All Pine Mortality Cases, Beetle-Related Mortality Cases, and Beetle-Related Advisory Cases with Seasonal Temperatures, Annual and Summer Drought Index (Precipitation minus Potential Evapotranspiration, (P-PET), and Tree-Ring–Based Mortality Index**

| | Temp. Spring | Temp. Summer | Temp. Fall | Temp. Winter | P-PET Aug.–July | P-PET May–Aug., Year 1 | Mortality Index |
|---|---|---|---|---|---|---|---|
| All mortality incidents | 0.40*** | 0.46*** | 0.42*** | 0.32*** | –0.08 | –0.12 | 0.30*** |
| Beetle-related mortality incidents | 0.46*** | 0.52*** | 0.36*** | 0.27*** | –0.07 | –0.13 | 0.26*** |
| Advisory cases | 0.29 | 0.34 | –0.18 | 0.05 | –0.29 | –0.39* | 0.72*** |

* *p* value < 0.1, ** *p* value < 0.05, *** *p* value < 0.01. The *p* values are only given for relative comparisons, but due to the temporal autocorrelation in the time series values, they must be used with caution.

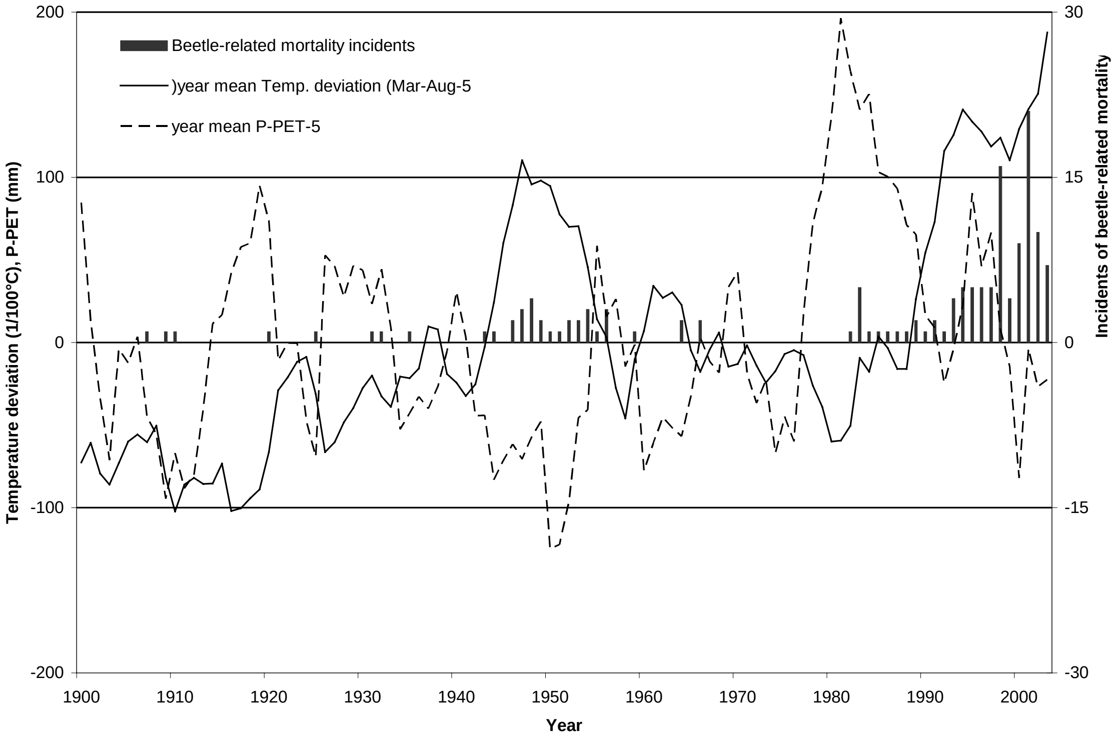

**FIGURE 5**. Incidents of beetle-related mortality and 5-year mean temperature deviation (March to August) and for annual drought index (precipitation minus potential evapotranspiration).

**TABLE 2**
**Percentage of All Felled Trees with One of the Three Main Beetle Species Emerging After a Dry or a Wet Summer and *p* Values of the Chi-Square Test for Differences between Dry and Wet Summers**

| Beetle Species | Dry Summer (2000, 2003, 2004) | Wet Summer (2001, 2002) | Chi-Square, *p* Values |
|---|---|---|---|
| *Phaenops cyanea* | 16.5% | 5.7% | 0.017 |
| *Tomicus* spp. | 12.6% | 4.6% | 0.056 |
| *Ips acuminatus* | 6.6% | 5.6% | 0.783 |
| All three above | 25.6% | 11.4% | 0.010 |

## DISCUSSION

Beetle-related mortality as described in forest reports have increased in recent years and coincided with periods of above-average temperatures. It seems very likely that higher temperatures have favored beetle development and thus increased population size and infestation pressure[15]. Warmer years also coincided with periods of drought that weakened trees as indicated in the tree-ring–based mortality index and observed mortality rates on the monitoring plot. However, during the dry, but colder, years in the early 1960s and 1970s, no increased bark beetle occurrence was noted. It may be possible that during the 1960s and 1970s, a period of high fluoride pollution from nearby aluminum smelters[16], reports focused mainly on air pollution rather than on beetle attacks. Also, changes in the regional Forest Service and more available recent reports may have influenced the reporting, and may have resulted in more reported mortality incidents in recent years, which may explain the relatively low correlation between beetle-related incidents from reports and beetle-related advisory cases.

Many studies have shown that summer drought increases subsequent tree mortality and reduces tree-ring width[17,18]. Advisory cases were highly correlated with tree-ring–based mortality index. Thus, it is highly likely that weakened trees allowed beetles to develop more successfully, eventually contributing to subsequent tree mortality[19]. Increased bark beetle levels in our felled trees following dry summers support this hypothesis. Tree mortality and beetle population seemed to be tightly coupled to water availability of previous summers. Physiological studies in the same area confirm that Scots pine closes its stomata earlier during dry and warm days and when water availability is low, while co-occurring downy oak is more drought and heat tolerant[20]. It is known that as a consequence of severe water stress, Scots pine reduces the length of the induced defense reaction and lowers the resin content[21,22], facilitating beetle infestations in the affected stands[23].

Other factors possibly affecting the vitality of the pines not considered in this study are air pollution, in particular ozone ($O_3$), nitrogen deposition, fluoride emissions, and forest use. While in the late 1970s fluoride emissions from near-by aluminum smelters caused extensive needle necrosis and were considered a main factor for Scots pine decline in the area[16], the introduction of emission-reduction technologies in the early 1980s drastically reduced the emissions and no further necrosis was reported[24]. Acidic deposition is less relevant, since almost all the soils developed from calcareous parent material. Modeled nitrogen deposition and the exceedance of its critical loads for forests in Switzerland show rather small values in the Valais as compared to the areas north and south of the Alps[25]. The relatively low total nitrogen deposition rates of less than 10 kg/ha/year found at the LWF site Visp with calculated critical loads of 10 kg/ha/year confirm these models (A. Thimonier, personal communicatoin). However, it may be possible that for sites with low productivity and limited water availability, the critical loads are overestimated by the standard models. Measured AOT40 $O_3$ concentrations in Visp in 2002 were below the values of most other long-term forest research sites in Switzerland, but critical levels were exceeded and symptoms on sensitive plants were present[26]. On the other hand, it is well known that, while $O_3$ concentrations are higher in warm and dry summers, $O_3$ uptake by plants is lower under water limitation[27]. We conclude, therefore, that while nitrogen deposition and $O_3$ concentrations may contribute to reduced tree vitality, they seem to be of low importance for the observed pine decline in Valais.

Forest management and use in the Valais, in particular thinning intensity, fuel wood collection, grazing, and litter raking, have all decreased or even subsided in recent decades, causing higher tree and shrub densities and thus more competition for water[28]. As pines have been found to react to increased competition with more stem growth reductions than oaks[29], this may be an indicator of higher susceptibility of pines to beetle attacks in recent years. In addition, the reduced removal of moribund or newly dead trees resulting in more potential breeding material for bark beetles may have further increased bark beetle densities. Thus, it can be assumed that it is likely that changed forest use has also contributed to increased beetle population density and perhaps higher pine mortality.

In conclusion, our data support the hypothesis that drought as an inciting stress factor weakens trees, whereas warmer spring and summer temperatures favor development of bark beetles, which contributes to the high regional Scots pine mortality[30]. Recent increase in temperatures coupled with several dry years since 1990 have led to unprecedented Scots pine mortality and may have increased population sizes of various beetles that are potentially harmful to stressed Scots pines. In other parts of the world recent drought and increased temperatures have also led to bark beetle outbreaks of much larger magnitude, such as the spruce bark beetle (*Dendroctonus rufipennis*) in Alaska[3], the mountain pine beetle (*Dendroctonus ponderosae*) in British Columbia[4], and various bark beetles on ponderosa and pinyon pine in the southwest of the U.S.[5]. However, it cannot be ruled out that air pollutants, such as high $O_3$ concentrations and nitrogen deposition have contributed to the pine decline by weakening pines, but their impact is considered as minor.

## ACKNOWLEDGMENTS

The study was financed by the Forest Service of the Canton Valais, the Federal Ministry for the Environment (BAFU), the Velux foundation, and the Swiss Federal Research Institute for Forest, Snow and Landscape Research WSL. We would like to thank the various field and laboratory technicians for their important work.
